# Vertically Integrated Silicon–Carbon Nanotube Architectures for High-Capacity and Robust Lithium-Ion Battery Anodes

**DOI:** 10.1021/acsaem.5c03862

**Published:** 2026-03-23

**Authors:** Muhammad Ahmad, Asim Mumtaz, Filipe Braga, Kai Yang, Peter Yates, Thomas P. Shalvey, Oliver S. Hutter, Matthew Bilton, Jonathan D. Major, Ken Durose, Laurence J. Hardwick, S. Ravi P. Silva

**Affiliations:** † Advanced Technology Institute, School of Computer Science and Electronic Engineering, 3660 University of Surrey, Guildford GU2 7XH, U.K.; ‡ Institute for Sustainability, University of Surrey, Guildford GU2 7XH, U.K.; § 152543 University of York, School of Physics, Engineering and Technology, Heslington, York YO10 5DD, U.K.; ∥ 4591 University of Liverpool, Stephenson Institute for Renewable Energy, Department of Physics, Peach Street, Liverpool L69 7ZF, U.K.; ⊥ 4591 University of Liverpool, Stephenson Institute for Renewable Energy, Department of Chemistry, Peach Street, Liverpool L69 7ZF, U.K.; # The University of Manchester, Nancy Rothwell Building, Booth Street East, Manchester M1 7HL, U.K.; ∇ Northumbria University, Newcastle Upon Tyne NE1 8QH, U.K.; ○ SEM Shared Research Facility, 105731 University of Liverpool, Brownlow Street, Liverpool L69 3GL, U.K.

**Keywords:** CVD grown carbon nanotubes, silicon−carbon nanotube hybrid structure, Li-ion battery anodes, carbon nanotubes on Cu foil, customized carbon nanotubes for battery anodes, high-capacity anodes

## Abstract

Lithium-ion batteries (LIBs) are going through a metamorphosis, progressing toward energy sources and storage that span wearables to grid-based storage; they are now moving toward bionic and space applications too. The performance of LIBs is often hindered by conventional anode materials, which suffer from restricted capacity, excessive volumetric expansion, dendrite formation, and an unstable solid electrolyte interphase (SEI) layer. This study introduces a breakthrough approach to fabricate vertically integrated silicon–carbon nanotube (VISiCNT) structures directly on copper foil. This architecture not only achieves exceptionally high capacities but also effectively accommodates volumetric expansion and mitigates material delamination. In addition, the high-quality growth of CNTs on copper foil is demonstrated at a rapid rate of 21 μm/min, suitable for roll-to-roll scale-up and large-scale manufacture. An unprecedented systematic investigation of various VISiCNT structural variants revealed that shorter CNTs (<5 μm) with a higher defect density (*I*_D_/*I*_G_ ≥ 1) deliver some of the highest reversible capacities, exceeding 3500 mAh g^–1^, albeit at low loadings, while also exhibiting good cyclic stability. This research delineates a clear pathway for the development of VISiCNT anode structures that combine exceptionally high capacity with enhanced cyclic stability, thereby providing valuable insights for advancing next-generation energy storage solutions.

## Graphical abstract

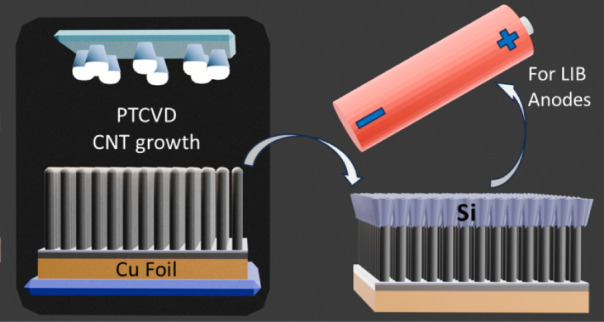

## Introduction

Electrical energy storage is vital for decarbonizing the grid and enabling the dispatchability of wind and solar power. Lithium-ion batteries (LIBs) are central to this transition, powering portable electronics and electrifying transport. The global LIB market is projected to grow from $117.8 billion in 2024 to $221.7 billion by 2029, with a CAGR of 13.5%. To meet the European Commission’s 2030 target of a 55% reduction in greenhouse gas emissions, LIBs must deliver ultrahigh energy and power density, longevity, and safety.^1^ Consequently, significant research focuses on enhancing key battery components such as electrolytes, separators, cathodes, and anodes.^2,3^ Graphite, the dominant anode material, is limited by its theoretical capacity of 372 mAh g^–1^.^4^ Among alternative materials, Si stands out due to its remarkable theoretical capacity of up to 4200 mAh g^–1^, low working potential (<0.4 V vs Li^+^/Li), cost-effectiveness, and abundance.^5–7^ However, Si suffers from severe volumetric expansion (300–400%) during lithium insertion and extraction, leading to pulverization, material detachment, and unstable solid-electrolyte interphase (SEI) formation, which degrades battery performance.^8,9^ Further, its low electrical conductivity (∼10^–3^ S cm^–1^) results in slow electrochemical kinetics, hindering fast charging/discharging, and its tendency for dendrite formation results in early failure of the batteries.^6,10,11^

CNTs present several advantages, including high surface area, superior electrical and thermal conductivity, lightweight properties, excellent tensile strength, and a Li^+^ storage capacity of up to 1116 mAh g^–1^, making them a viable anode candidate.^8,12–15^ However, their large first-cycle loss, absence of a voltage plateau, and formation of an unstable solid-electrolyte interphase (SEI) layer render them suboptimal.^13,16,17^ To derive performance, CNTs are primarily used as additives to graphite and Si composites to enhance reversibility and charge transport efficiency.^13,18,19^ This requires harsh chemical treatments (acids and surfactants) for dispersion or involves mechanical processing, such as ball milling, which compromises their intrinsic properties.^18–20^ Additionally, random dispersion and alignment issues reduce their effectiveness in the charge transport process. Given their anisotropic electrical conductivity, ensuring direct contact of individual CNTs with the Cu current collector is critical for achieving optimal battery performance.^21,22^ Furthermore, CNT performance is dependent on various parameters such as length and structural quality, which are difficult to control in solution-based processes.

CNTs grown directly on Cu foil via chemical vapor deposition (CVD) provide a promising solution, ensuring superior structural integrity, eliminating the need for polymeric binders, and tailoring critical CNT parameters (length, density, diameter, and metallicity).^8,23^ Despite significant advancements, the direct growth of CNTs on conductive substrates remains a technical challenge.^24,25^ Existing literature predominantly reports CNT synthesis on rigid and insulating inorganic materials, such as Si, SiO_2_, and Al_2_O_3_, at elevated temperatures exceeding 600 °C.^26^ In many cases, CNTs must subsequently be transferred from these substrates to conductive surfaces, adding complexity to the fabrication process.^27,28^ *In situ* CNT growth on Cu foil, a promising approach for LIB anodes, remains relatively unexplored. There are few reports detailing direct CNT synthesis on Cu foil, and even fewer studies investigating its application as an LIB anode material.^8,29,30^

Several studies have explored CNT-Si composites to enhance LIB anode performance.^5,31–33^ Cui et al. synthesized free-standing CNT films on stainless-steel mesh from an aqueous solution, followed by Si coating via silane-based CVD, resulting in a specific capacity of ∼2000 mAh g^–1^ with stability over 50 cycles.^34^ Gonzalez et al. synthesized Si-doped CNTs in powder form using a modified CVD system, employing toluene, ferrocene, and triphenylsilane as carbon, catalyst, and Si sources, respectively, and obtained a reversible capacity of ∼400 mAh g^–1^.^35^ Wang et al. sputtered Si onto cone-shaped graphene-CNT clusters synthesized using chemical vapor deposition (CVD) at 750–1000 °C, reporting an initial reversible capacity of 1954 mAh g^–1^, which decreased to 1200 mAh g^–1^ after 250 cycles.^36^ Zhang et al. developed porous Si microparticles/CNT composites using a ZnCl_2_-assisted molten salt dealloying process combined with acid treatment, yielding a reversible capacity of 797 mAh g^–1^.^10^ Nguyen et al. fabricated a self-standing negative electrode by dispersing CNTs, Si, and graphene oxide in an aqueous solution, followed by filtration, achieving 2342 mAh g^–1^.^37^ Ikonen et al. conjugated positively charged (−NH_2_) single-walled CNTs (SWCNTs) with negatively charged (−COOH) thermally carbonized mesoporous Si microparticles, further treated with succinic anhydride (SA), achieving their best anodic capacity of 1150 mAh g^–1^ over 110 cycles at a 0.2 C rate.^18^ Recently, Hoseini et al. demonstrated MWCNT growth on stainless steel via plasma-enhanced CVD (PECVD), followed by Si deposition using DC sputtering, achieving 3250 mAh g^–1^ with strong cyclic stability.^31^ Despite these advancements, most reported Si-CNT anodes are fabricated using solution-based methods, ball milling techniques, or CNTs grown on substrates other than Cu foil, each presenting inherent limitations. Addressing these challenges by optimizing CNT growth directly on Cu foil for LIB anode applications remains an area of critical research.

In this study, we developed a vertically integrated silicon–carbon nanotube (VISiCNT) hybrid structure as a high-performance anode material, conducting a systematic investigation of CNTs with varying lengths and structural qualities grown directly on Cu foil, followed by Si coating. The high-quality growth of CNTs on Cu foil is demonstrated at a rapid rate of 21 μm/min using a photothermal chemical vapor deposition (PTCVD) system, a technique with the potential for scale-up through roll-to-roll production. Within the VISiCNT architecture, CNTs serve as highly conductive pathways, facilitating charge transport, enhancing surface area for Si attachment, accommodating Si expansion/contraction, and improving adhesion to the Cu current collector. The anode was evaluated in a half-cell configuration, demonstrating a synergistic effect between Si and CNTs, resulting in high anodic capacity values (>3500 mAh g^–1^), approaching the theoretical lithium-ion storage capacity of Si and good cyclic stability. The observed enhancement in capacity and cyclability is attributed to the presence of shorter (<5 μm) and defective CNTs in the structure. The VISiCNT structure presents a promising solution not only for integrated microsized, high-energy-density LIBs for microelectronics but also for large-scale production for applications including electric vehicle (EV) batteries and grid-scale energy storage systems

## Method

The VISiCNT structure for lithium-ion battery (LIB) anodes was fabricated by growing CNTs directly on Cu foil using the PTCVD system, followed by sputtering a Si layer over the CNTs, as schematically illustrated in Figure 1.

**Figure 1:**
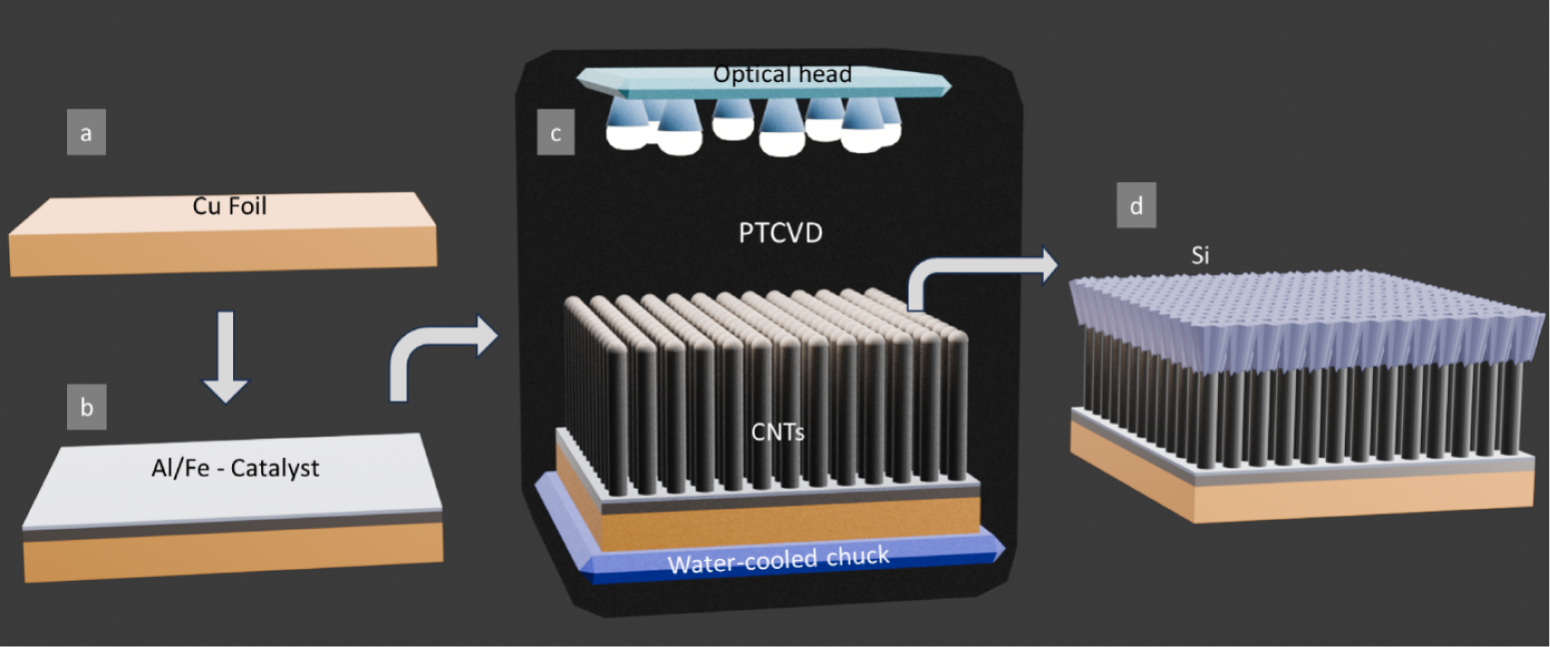
Schematic of process flow to realize VISiCNT structure for LIB anodes: (a) Cu foil, (b) coating Al/Fe (10/3 nm) as a catalyst on Cu foil, (c) PTCVD growth of CNTs directly on Cu foil, and (d) sputtering of Si on *in situ*-grown CNTs.

### CNT Growth on Cu Foil via PTCVD

A bilayer consisting of 10 nm of Al and 3 nm of Fe was first deposited on a Cu foil (Figure 1a,b) to serve as the catalyst for CNT growth. CNT synthesis was then conducted at substrate temperatures below 415 °C using the PTCVD system (Figure 1c).^24,25,28^ In contrast to conventional hot-wall CVD systems, the PTCVD employs a top-down heating arrangement through an array of eight optical lamps (each rated at a maximum of 1 kW), while the sample is held on a water-cooled chuck to maintain a low bulk temperature (Figure 1c).^38^ This configuration enables direct heat transfer at the CNT growth front while keeping the overall substrate temperature low. Temperature is monitored using a pyrometer positioned at the backside of the sample, ensuring an accurate measurement of the bulk substrate temperature. The PTCVD system was developed at the University of Surrey and was used in the creation of VANTABLACK, one of the world’s darkest synthetic materials, which is produced by Surrey NanoSystems (vantablack.co.uk). CNT growth proceeds by first catalyst reduction via heat treatment under flowing H_2_ (100 sccm) at 2  Torr, followed by the introduction of acetylene (C_2_H_2_) as the carbon precursor.

### Silicon Deposition by RF Sputtering

The RF sputtering of Si (Figure 1d) was carried out at room temperature under a chamber pressure of 3 mTorr using an argon (20 sccm) atmosphere. A high-purity (99.999%) undoped 2 inch silicon target was employed. With a power density of 0.66 W·cm^–2^, a deposition rate of 0.13 Å/s was achieved. Initial depositions on glass substrates were used to verify the thickness and deposition rate, measured by profilometers (Ambios XP–200 and Dektak XTL).

### Sample Sets and CNT Parameter Variation

Two sets of samples, differing in CNT length, structural quality, and silicon coating thickness, were prepared.

### Set One

A bilayer of Al (10 nm) and Fe (3 nm) was sputtered onto four Cu foil samples (∼2 × 3 cm^2^, ∼ 10 μm thick) using the JLS-MPS-500 magnetron sputter system (Shaded Green in Table 1). CNTs were synthesized at an approximate temperature of 415 °C (3.6 kW, corresponding to 45% lamp intensity), targeting lengths of 150 μm, 15 μm, and 3 μm. The sample aimed at producing 150 μm CNTs underwent an 8 min heat treatment, followed by 7 min of growth using 50 sccm C_2_H_2_. In contrast, the samples intended for 15 μm and 3 μm CNTs were subjected to a 10 min heat treatment and subsequent CNT growth using 10 sccm C_2_H_2_ for durations of 5 and 1 min, respectively. A 200 nm amorphous Si layer was deposited onto the 15 μm CNT sample and one of the 3 μm CNT samples, hereafter referred to as 15CNTs/Si and 3CNTs/Si, respectively. No Si coating was applied to the 150 μm CNT sample or the remaining 3 μm CNT sample, designated as 150CNTs and 3CNTs. The samples were cut into 12 mm diameter discs.

**Table 1:**
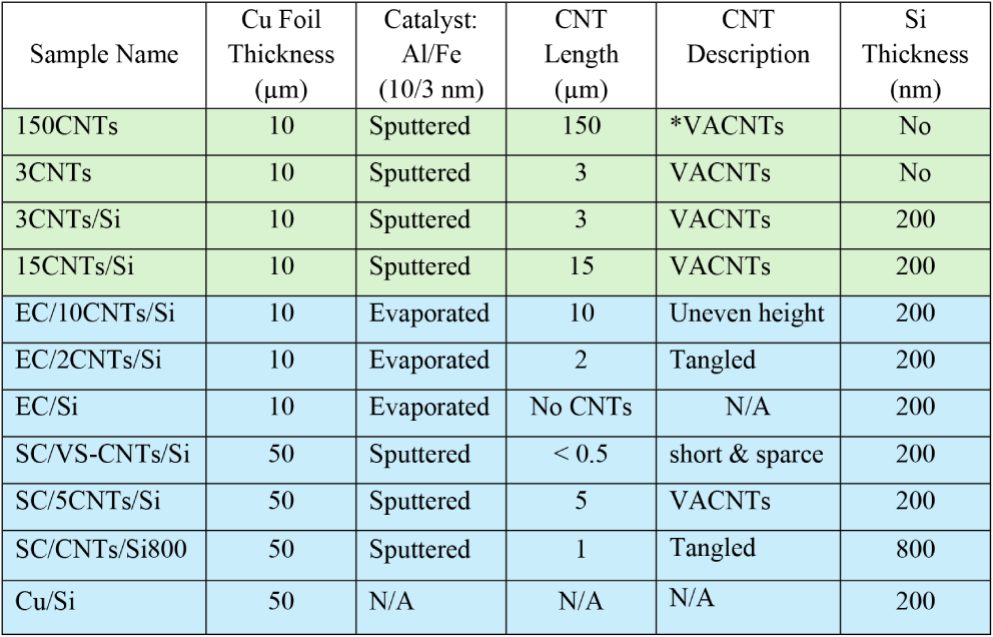
Sample Names and Variations^a^ ^b^

a Green-shaded samples are from Set one, and blue-shaded ones are from Set two.

b *VACNT: vertically aligned carbon nanotubes; EC: evaporated catalyst; SC: sputtered catalyst.

### Set Two

Seven samples were prepared using Cu foils of 10 μm and 50 μm thickness, each precut into discs of 12 mm diameter (Shaded Blue in Table 1). A catalyst comprising 10 nm Al and 3 nm Fe was deposited via either evaporation or sputtering. The evaporated catalyst was applied to three of the 10 μm thick Cu foils by using a Univex e-beam evaporator, while the sputtered catalyst was deposited onto three of the 50 μm thick Cu foils by using a JLS sputter system. An additional 50 μm Cu foil sample was directly sputtered with a 200 nm Si layer and designated as Cu/Si. CNTs were grown at 375 °C (40% lamp power) using a 10 min heat treatment and 10 sccm C_2_H_2_ flow. For the evaporated catalyst samples, CNTs with lengths of ∼10 μm and ∼2 μm were grown with growth times of 5 min and 1 min, respectively. No CNTs were grown on the third evaporated catalyst sample. A 200 nm Si layer was then sputtered on all three evaporated samples, yielding the following designations: EC/10CNT/Si, EC/2CNTs/Si, and EC/Si.

For the sputtered catalyst samples, very short (<500 nm) and sparse CNTs, 5 μm-long CNTs and 1 μm-long CNTs are grown using 15 s, 2 min, and 45 s growth times, respectively. A 200 nm Si layer was sputtered on the very short and 5 μm CNT samples, while an 800 nm Si layer was deposited on the 1 μm CNT sample. These samples are designated as SC/VS-CNT/Si, SC/5CNT/Si, and SC/CNT/Si800, respectively.

### Electrochemical Cell Assembly and Characterization

The fabricated samples were assembled into half-cells (CR2025 coin cells) in an argon-filled glovebox using Li foil discs as counter electrodes. The electrolyte consisted of a 45:45:10 volumetric mixture of ethylene carbonate (EC), dimethyl carbonate (DMC), and fluoroethylene carbonate (FEC), with FEC constituting 10% by volume; 100 μL of the electrolyte was added per cell to ensure complete coverage. Standard coin cell components, including a glass fiber separator and stainless-steel casing, were utilized. Galvanostatic cycling was performed within a voltage range of 0.01–1.5 V using a multichannel Maccor battery test system, following a protocol of 2 formation cycles at C/20 and 200 charge/discharge cycles at C/5. Electrochemical impedance spectroscopy (EIS) was conducted on cycled cells over a frequency range from 1 MHz to 1 Hz using a Biologic potentiostat system. The samples were characterized at various stages using SEM (Tescan Mira, FEI Helios Nanolab 600i) for surface morphology analysis and cross-sectional imaging, and Raman spectroscopy (Horiba XploRA PLUS MicroRaman Spectrometer, 532 nm laser) for evaluating the structural integrity and quality. Sample weights were also monitored at various stages using a Mettler Toledo microbalance (capacity: 22 g; readability: 0.002 mg). The method of accurately measuring the mass loading of ultralightweight active materials in electrodes is given in Supporting Information (SI).

## Results and Discussion

Figure 1 schematically illustrates the fabrication process of VISiCNT structures, which involves the growth of CNTs directly on a Cu foil following catalyst deposition, followed by Si sputtering onto the CNTs. SEM images and Raman spectra of CNTs grown on Cu foil and VISiCNT structures for the first set of samples are shown in Figure 2, and Figures S1 and S2 of Supporting Information (SI). Figure 2a displays an SEM image of the 150CNTs sample, which exhibits vertically aligned CNTs with heights of approximately 150 μm and diameters ranging between 10 and 20 nm, as estimated by high-magnification SEM images. Figure 2b,c provides micrographs of the Si-coated samples (3CNT/Si and 15CNT/Si), where the 200 nm silicon remains deposited atop the CNTs. Notably, the deposition does not penetrate into the densely packed CNT forest (approximately 10^9^ tubes/cm^2^), as schematically illustrated in Figure 2c. The growth rates are markedly different: the CNTs of the 150CNTs sample grow at a rate of approximately 21 μm/min, whereas the CNTs of the 15CNT/Si sample grow at about 3 μm/min.

**Figure 2:**
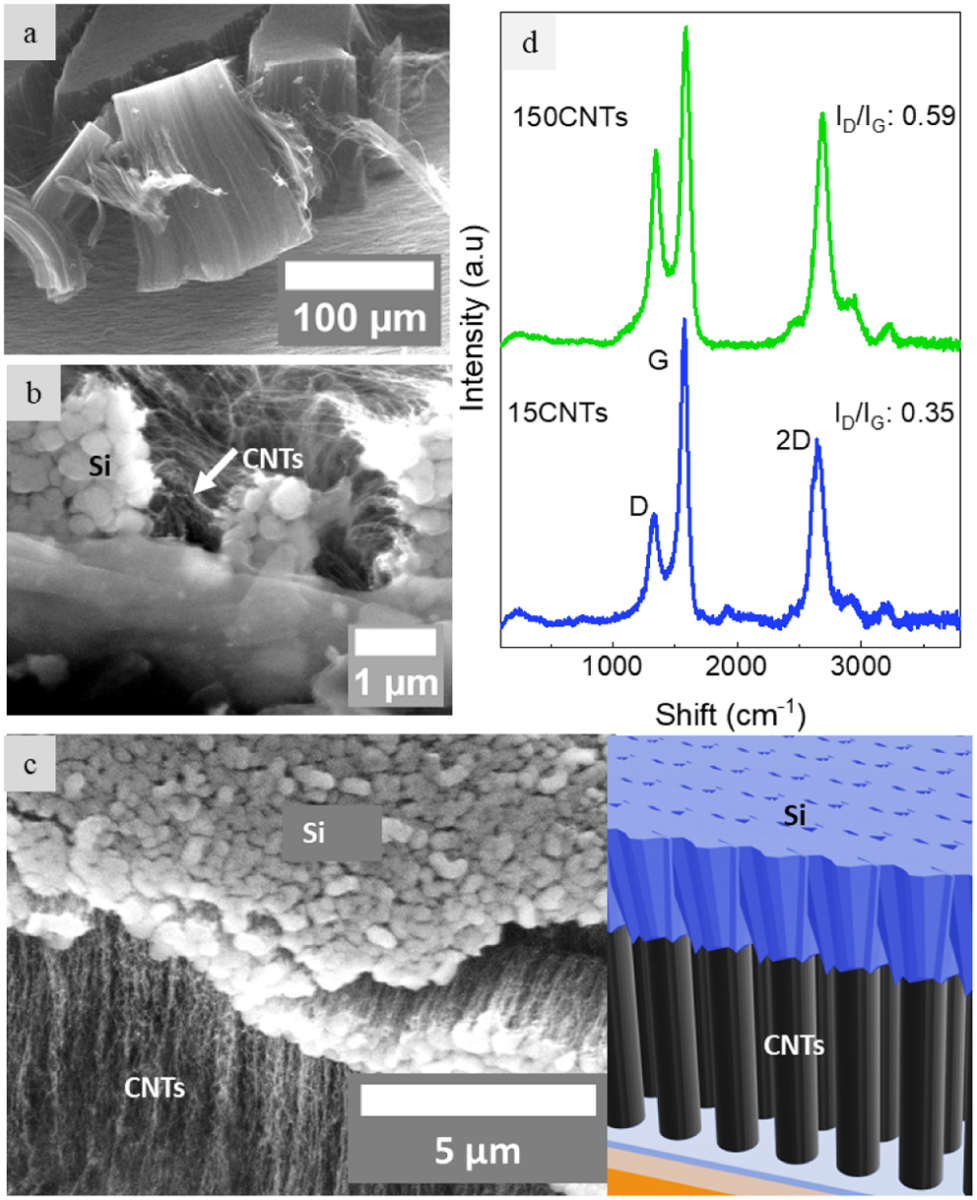
SEM images and Raman spectra of VISiCNT structures. (a) SEM image of the 150CNTs sample showing ∼150 μm tall forest of vertically aligned CNTs. (b) SEM image of the 3CNTs/Si sample showing 200 nm Si deposited on top of CNTs. (c) SEM image of the 15CNTs/Si sample showing 200 nm Si sputtered on CNTs. Adjacent is a schematic illustration of silicon remaining atop the CNTs. (d) Raman spectra of as-grown CNTs of 150CNTs and 15CNTs samples showing growth of high-quality CNTs with well-defined D-peaks around 1340 cm^–1^, G-peaks around 1580 cm^–1^, and 2D-peaks around 2700 cm^–1^. The D-peak is associated with defects, the G-peak indicates graphitization, the 2D peak is overtone of the D peak but independent of defects, and the intensity ratio of the D and G peaks is used to estimate structural quality of the material. The Low *I*_D_/*I*_G_ values of 0.59 and 0.35 indicate high structural quality of CNTs.

The Raman spectra in Figure 2d reveal distinct peaks: the D-peak at 1340 cm^–1^, the G-peak at 1580 cm^–1^, and the 2D-peak at 2700 cm^–1^. The D-peak is attributed to disorder-induced A_1g_ modes arising from structural defects in the hexagonal carbon lattice, with its intensity correlating with the defect density.^39^ In contrast, the overtone 2D-peak is largely unaffected by these defects since both phonon-assisted scattering events are involved.^40^ The G-peak, corresponding to the Raman-active E_2g_ vibrational mode of sp^2^-bonded carbon atoms, serves as a measure of the degree of graphitization.^41^ The intensity ratio (*I*_D_/*I*_G_) is commonly used as an indicator of material quality, with lower ratios reflecting higher structural quality.^42^ In this study, *I*_D_/*I*_G_ values of 0.59 (for 150CNTs) and 0.35 (for 15CNTs/Si) demonstrate the production of high-quality CNTs at 415 °C, comparable to those grown at temperatures exceeding 700 °C via conventional methods.^43–45^ Figure S3 represents the Raman spectrum of Si sputtered on CNTs, displaying a broad band centered at 480 cm^–1^, which is characteristic of amorphous silicon.^46^

The improved quality (*I*_D_/*I*_G_ of 0.35) observed for the 15CNTs/Si is attributed to the reduced C_2_H_2_ flow rate (10 sccm) during synthesis, in contrast to the 50 sccm used for the 150CNTs.^47^ However, increasing the C_2_H_2_ flow rate resulted in a markedly accelerated CNT growth rate of approximately 21 μm/min, yielding a ∼150 μm tall forest within just 7 min. This rapid synthesis method is well-suited for roll-to-roll, large-scale production and demonstrates extensive applicability across diverse domains, including batteries, CNT wires, nonmetallic motors, robotics, composite materials, aerospace, and electronic devices. To our knowledge, this work represents the highest reported CNT growth rate on thin copper foil (10 μm) at temperatures below 450 °C while still achieving an adequate structural quality (*I*_D_/*I*_G_: 0.59).

In contrast, previous studies have typically relied on CNT growth at temperatures above 700 °C using thicker Cu foils (e.g., 50 μm).^8,29,30,48,49^ Such approaches contribute unnecessary cost and weight and ultimately reduce the specific capacity of Li-ion battery cells. For instance, Lahiri et al. reported the direct growth of randomly oriented CNTs on 50 μm Cu foil using Ti and Ni catalysts (20–25 nm) at temperatures between 700 and 900 °C, yielding CNTs approximately 30 μm in height with diameters around 100 nm and a lower structural quality (*I*_D_/*I*_G_ ≈ 1).^8^ Commercial Cu foils for anode current collectors generally range between 6 and 20 μm in thickness, and in the context of a 90 kWh Tesla Model S battery, Cu accounts for roughly 0.334 kg/kWh (≈30 kg total).^34,50^ Li et al. demonstrated CVD growth of CNTs on 0.062 in. (∼1.6 mm) thick Cu alloy, resulting in 30 μm height and *I*_D_/*I*_G_ value of 1.1, using a mixture of Ar, H_2_, and C_2_H_4_ through a water bubbler (water vapor) at 700 °C.^48^ Wang Wei et al. reported an interesting hybrid structure of graphene/CNTs grown on 20 μm thick Cu foil using a two step ambient CVD process, where CH_4_/H_2_ is used for graphene growth at 950 °C in the first step and then C_2_H_4_/H_2_ for CNT growth at 750 °C after depositing a thin layer of Fe as a catalyst.^30^ Vertically aligned CNTs with a 30–40 μm estimated height and *I*_D_/*I*_G_ value of ∼0.91 can be observed in the SEM images and Raman spectra provided in their paper. A similar approach was adopted by Raji et al., who grew graphene-CNT structures on 25 μm Cu foil at 1000 °C temperature and performed Li plating for 3 h before cell assembly.^49^ Lettiere et al. observed a degradation in CNT structural quality (*I*_D_/*I*_G_ increasing from 0.53 to 1) when forest height exceeded 7.5 μm.^29^ They used a multilayer catalyst stack consisting of W, Al_2_O_3_, and Fe deposited on a 35 μm thick Cu foil and grew CNTs using atmospheric-pressure CVD in a tube furnace at 750 °C. The process employed a He/H_2_/C_2_H_4_ gas mixture and involved a complex multistep sequence lasting more than 50 minutes, producing CNTs with heights ranging from 7.5 to 270 μm. In contrast, our CNTs, displaying *I*_D_/*I*_G_ ratios of 0.35 for 15 μm and 0.59 for 150 μm heights, are significantly superior in terms of structural quality and growth rate compared with these literature reports.

The specific capacity results obtained from half-cell assemblies, along with postcycling SEM images, are shown in Figure 3 and Figure S4. As shown in Figure S4a, the initial formation cycle of the 150CNTs sample exhibits a considerably higher capacity compared to the 3CNTs sample. In contrast, the capacities of the Si-coated CNT samples (3CNT/Si and 15CNT/Si) are comparable (approximately 0.25 mAh cm^–2^) despite differences in CNT lengths (3 and 15 μm), which indicates the dominant role of silicon in Li^+^ storage. The prolonged voltage plateau for 150CNTs is attributed to their larger surface area.^51^ However, the capacity provided by pure CNT samples is largely nonreversible; this is evidenced by a differential capacity peak near 0.9 V (Figure S1b), which indicates the formation of an unstable solid electrolyte interphase (SEI) layer that irreversibly consumes the electrolyte.^17,51,52^ Although the reversible capacity of pure CNTs is much lower than that of Si-coated CNTs, they still exhibit measurable and stable electrochemical performance. In particular, the 150CNTs sample delivers a capacity of approximately 150 mAh g^–1^ with no observable capacity fade over 200 cycles. This performance is consistent with some of the reported values for pure CNT anodes in the literature, where capacities typically range from 80 mAh g^–1^ to 250 mAh g^–1^.^51,53^

**Figure 3:**
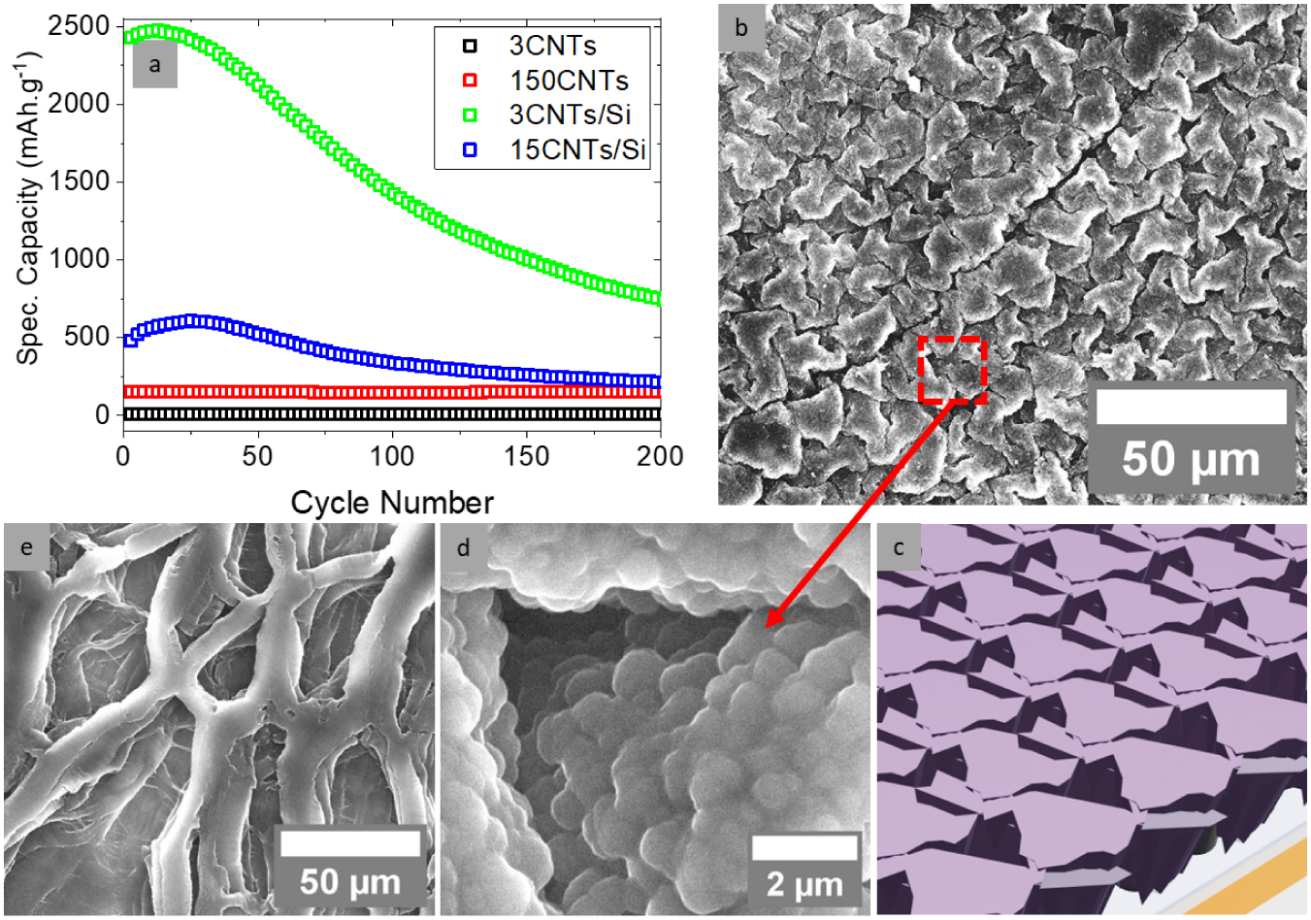
Cyclic capacity of the samples tested in half-cell assembly and their postcycling SEM images. (a) Specific capacities showing very little capacity for pure CNT samples (150CNTs and 3CNTs) but significantly higher capacities for VISiCNT structures (3CNT/Si and 15CNT/Si). (b–e) SEM images of the postcycled samples: (b, c) SEM image and schematic illustration of the 3CNTs/Si sample showing cracking of the Si film but no material detachment. (d) High-magnification SEM image of the 3CNT/Si sample. (e) The image shows that the CNTs in the 150CNTs sample are deformed due to the cell assembly and densification effect of CNTs, but no removal of CNTs was observed.

Figure 3a details specific cyclic capacities over 200 cycles: the Si-coated samples exhibit capacities of 2460 and 580 mAh g^–1^ at cycle 15 for 3CNT/Si and 15CNT/Si samples, respectively, which significantly outperform the pure CNT samples (3CNT, 150CNT). Additionally, the larger mass of the 15CNT/Si sample (0.168 mg versus 0.06 mg for 3CNT/Si) contributes to its lower specific capacity. Interestingly, the storage capacity of the VISiCNT structures increases over the first 25 cycles, a phenomenon attributed to the progressive fragmentation of silicon (electrochemical milling), which facilitates enhanced lithium alloying, before subsequently decreasing in later cycles.^54^

Figure 3b–e and Figure S5 display SEM images of the electrodes following cycling. Both the pure CNT and the VISiCNT samples exhibit excellent structural integrity and adherence to the Cu foil (Figure 3b–d). Although the vertical alignment of CNTs in the 150CNTs sample (see Figure 3e) is somewhat deformed due to the compression from cell assembly and the densification caused by contact with liquid electrolyte, the overall material attachment remains robust.^30,55^ Moreover, the observed increase in nanotube diameter, from an initial 10–20 nm to approximately 60–80 nm, suggests the formation of a solid electrolyte interphase (SEI) layer, which is associated with the irreversible consumption of Li ions.^8,30^ Notably, even in regions where cracks develop (Figure 3b–d), the silicon remains firmly adhered to the CNTs, preserving both physical and electrical connection to the Cu substrate. This strong interfacial bonding contributes to the higher capacity and cyclic stability of the cells by preventing the detachment of active material during the volumetric changes experienced in lithiation and delithiation cycles.

Interestingly, and contrary to popular belief, the performance of VISiCNT structures improved with shorter CNTs relative to longer ones (Figure 3a). To further investigate this phenomenon, we prepared a second set of samples featuring shorter CNTs (ranging from submicron to 10 μm) grown on Cu foils. Representative SEM images and Raman spectra for selected samples after CNT growth are presented in Figure S6 of SI. The morphology of CNT growth on the EC/10CNTs/Si sample (Figure S6a) is markedly different from that observed in the other samples. In this sample, CNT bundles exhibit uneven heights ranging from 5 to 15 μm. In contrast, the SC/5CNTs/Si sample displays vertically aligned CNTs of around 5 μm in length (Figure S6b). This difference arises from the use of evaporation versus sputtering for catalyst deposition, as these techniques produce films with fundamentally different microstructures due to the distinct energy and trajectories of the arriving atoms. Sputtering typically yields dense, compact films with fewer voids and stronger adhesion, whereas evaporation tends to produce relatively porous, columnar structures because of its lower-energy, line-of-sight deposition nature.

Furthermore, the SC/VS-CNTs/Si sample shows very short (<500 nm) and sparse CNTs (Figure S6c), along with carbon-encapsulated catalyst particles, as schematically illustrated in the inset of the figure. Figure S6d shows the SEM image of the SC/CNTs/Si800 sample, where tangled CNTs of approximately 1 μm in length are coated with 800 nm of silicon. The Raman spectrum of the SC/VS-CNTs/Si sample features poorly defined D and G peaks, in contrast to the more pronounced peaks observed for the EC/10CNTs/Si and SC/5CNTs/Si samples. The higher *I*_D_/*I*_G_ ratios (1.0 and 1.11) in these samples indicate a greater density of structural defects, which is a consequence of deliberately selecting a lower growth temperature (375 °C) for the CNT synthesis.^25,28^

Figure 4 presents plots of specific capacities, decay rates, areal capacities, Coulombic efficiencies, and EIS analysis for the second set of samples. Figure 5 presents SEM images of selected samples before (left column) and after cycling (right column). In Figure 4a, the specific capacities are plotted with black dashed lines representing linear fits between cycles 15 and 198 (the corresponding capacity values are provided in Table S3 of the SI). The slopes of these linear fits are used to derive the capacity decay rate. Notably, the EC/2CNTs/Si and SC/VS-CNTs/Si samples exhibit exceptionally high capacities of 3545 mAh g^–1^ and 3580 mAh g^–1^, respectively; among the best reported in the literature and approaching the theoretical capacity of silicon.^10,12,36,54,56^ These capacity values exceed those reported by Raji et al. for Li-plated, CVD-grown graphene–CNT structures fabricated at 1000 and 750 °C on 25 μm Cu foil.^49^ Our anode architecture is advantageous because it utilizes low-cost, CMOS-compatible, earth-abundant, and safely processable materials (silicon and carbon), in contrast to the relatively costly and demanding lithium-based negative electrodes.

**Figure 4:**
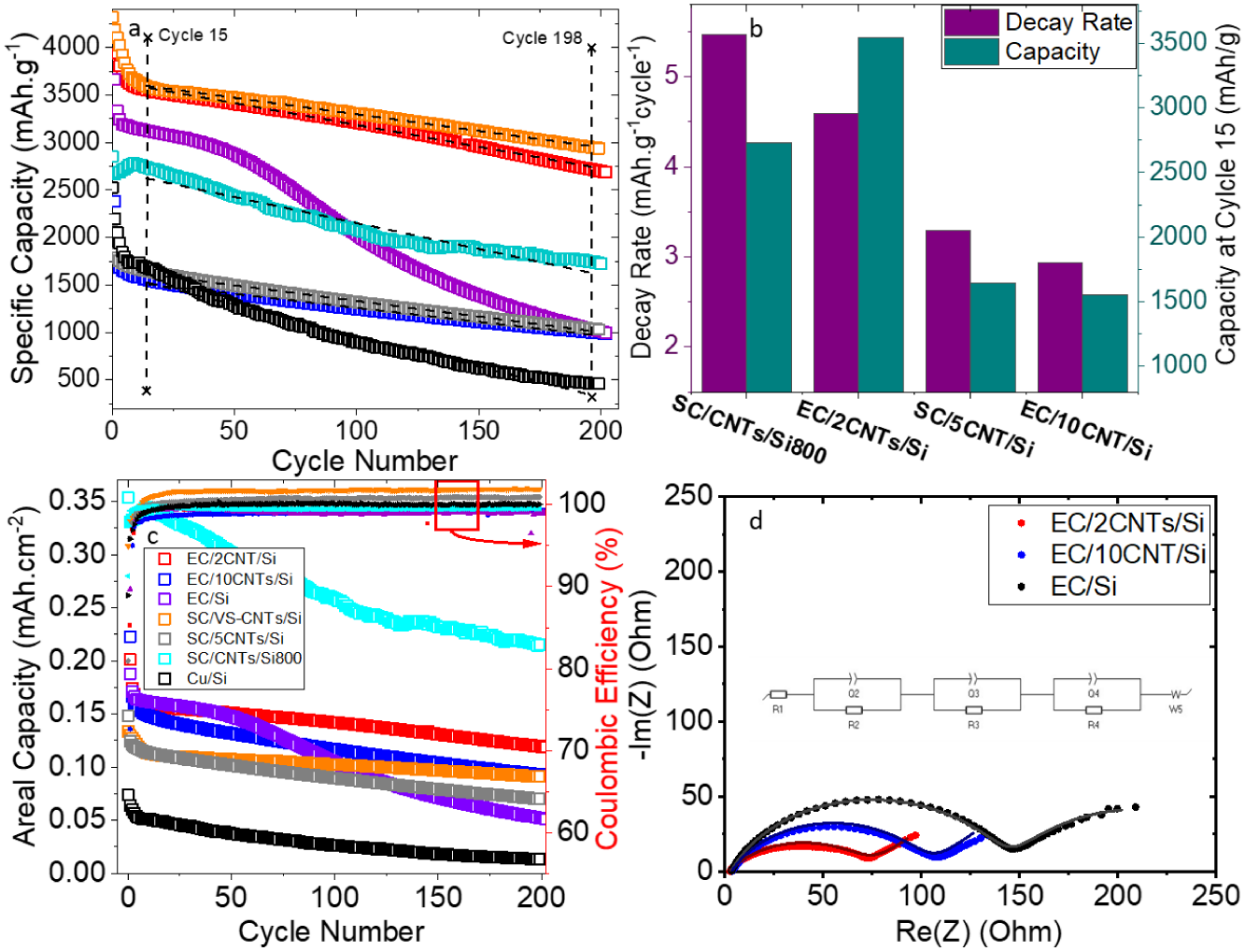
Anode testing and characterization results of VISiCNT structures in the set two of samples. (a) Specific capacities of the samples, showing one of the highest capacities above 3500 mAh g^–1^ for EC/2CNTs/Si and SC/VS-CNTs/Si samples. The figure legend of (c) also applies to (a). (b) The decay rate and capacity of the samples, showing a slower decay rate for longer CNTs and higher capacity for shorter CNTs of VISiCNT structures. (c) Areal capacities (left-side *y*-axis and Coulombic efficiencies (right-side *y*-axis; curves in red squares) of the samples. Coulombic efficiencies of the samples at cycle 15 show values above 98%. (d) Nyquist plot (EIS) from 1  MHz to 1  Hz of selected samples after cycling, where samples containing CNTs (EC/2CNTs/Si, EC/10CNTs/Si) exhibit lower charge transport resistance compared with the EC/Si sample where no CNTs are present.

**Figure 5:**
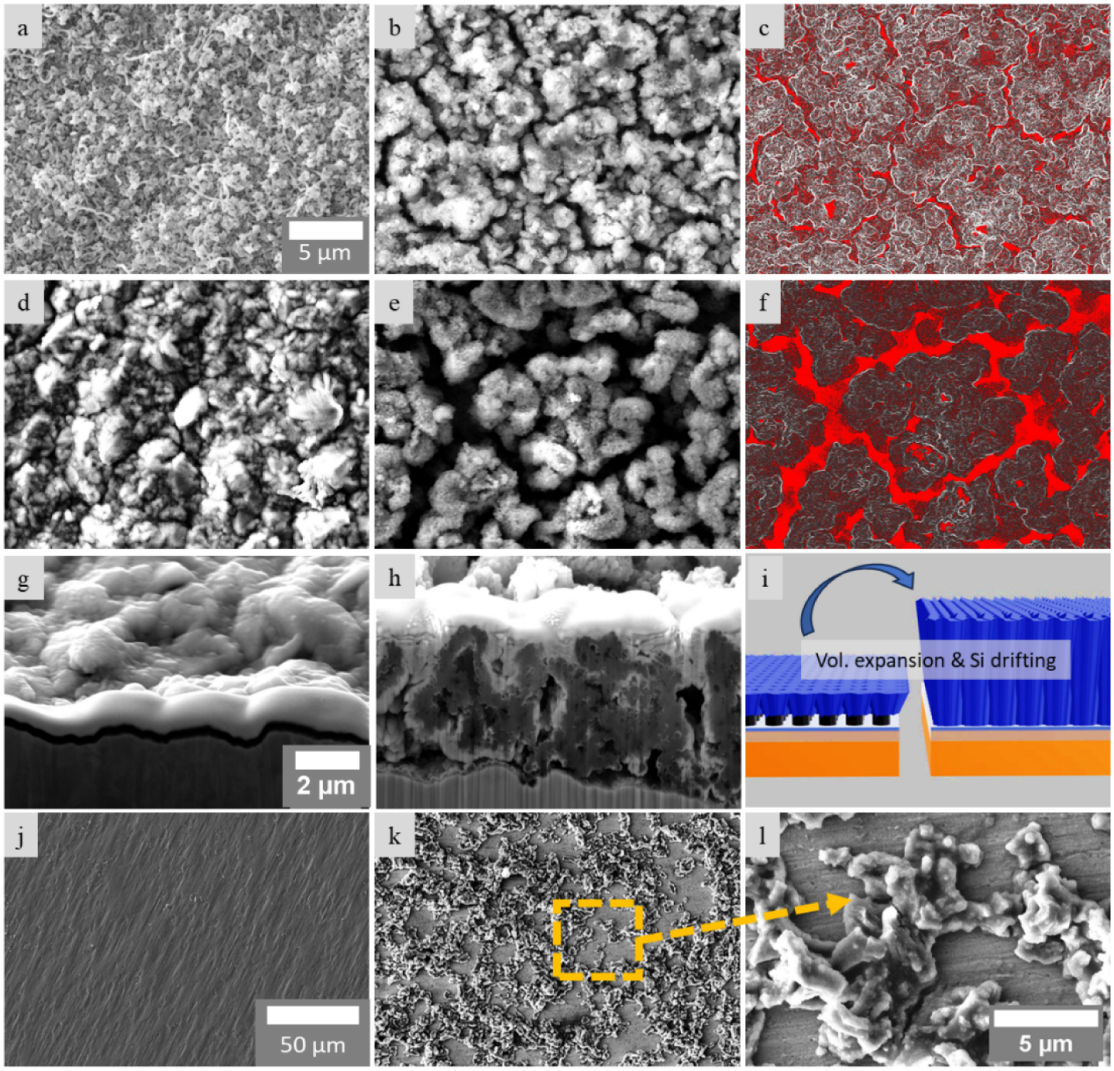
SEM images of the second set of samples before and after cycling. (a,b) SEM image of the EC/10CNTs/Si sample before and after cycling, respectively, showing Si pulverization and crack development after cycling. (c) The crack area of the EC/10CNTs/Si sample analyzed and calculated to be ∼19% using ImageJ. (d, e) SEM images of EC/2CNTs/Si samples before and after cycling. (f) The crack area is estimated to be ∼38% using ImageJ. (g, h) Cross-section SEM images of EC/2CNTs/Si before and after cycling showing volumetric expansion of ∼ 400% after cycling. (i) Schematic illustration of the volumetric expansion and Si drifting. (j,k) SEM image of the EC/Si sample before and after cycling showing material delamination after cycling. (l) High-magnification SEM image of (k). Scale bars on (a–f), (g,h), and (j,k) are equal.

Both the EC/2CNTs/Si and SC/VS-CNTs/Si samples contain short CNTs (2 μm and <0.5 μm, respectively), corroborating our earlier observation (Figure 3a) that shorter CNTs in the VISiCNT structure perform better than their longer counterparts. The shorter CNTs contribute to a lower overall electrode weight and minimize irreversible Li consumption due to stable SEI formation while still providing sufficient electrical conductivity and mechanical support to the silicon. Consequently, higher specific capacities are achieved. In particular, the specific capacity of the SC/VS-CNTs/Si sample is marginally higher than that of the EC/2CNTs/Si sample due to the negligible weight contribution from the very short and sparse CNTs (as evidenced in Figure S6c). Furthermore, the carbon-encapsulated catalyst particles observed in this sample may enhance adhesion and better accommodate the volumetric changes in silicon during lithiation and delithiation cycles.

In contrast, the longer CNT samples (EC/10CNTs/Si and SC/5CNTs/Si) exhibit considerably lower specific capacities at cycle 15 (1550 and 1640 mAh g^–1^, respectively). This reduction is primarily attributed to the increased mass contribution of the longer CNTs and enhanced irreversible lithium consumption resulting from the SEI formation around CNTs.^51^ These results imply that within the VISiCNT structure, the CNTs mainly provide structural stability and facilitate rapid charge exchange due to their high conductivity, rather than directly contributing to the lithiation/delithiation process. This observation underscores the importance of optimizing the CNT length; excessively long CNTs not only add redundant weight but also exacerbate capacity degradation through the formation of an unstable SEI.

The SC/CNT/Si800 sample, which features an 800 nm thick Si layer on approximately 1 μm long CNTs, achieves a specific capacity of 2725 mAh g^–1^ at cycle 15 and demonstrates good cyclic stability, maintaining capacities above 1700 mAh g^–1^ after 200 cycles. This is particularly notable considering that thicker Si films typically suffer from rapid capacity decay.^54,57^ Interestingly, the sample containing only the catalyst (EC/Si) initially displays high specific capacities for the first 50 cycles, which can be attributed to the enhanced adhesion provided by the Al/Fe catalyst layers on the Cu foil. This behavior is analogous to a previous report where thin Si films (50–150 nm) evaporated on Ni foils exhibited high capacities and extended cyclability.^54^ In our case, however, the catalyst layers are unable to sustain adequate adhesion beyond 50 cycles; subsequent Si pulverization leads to detachment from the Cu foil (as shown in Figure 5f), resulting in a sharp capacity decline. Furthermore, the control sample (Cu/Si), which contains neither CNTs nor catalyst, shows a similarly high initial capacity to the Si-coated CNT samples (1674 mAh g^–1^) but suffers from a pronounced capacity fading rate of 6.51 mAh g^–1^ per cycle, resulting in a low capacity of 460 mAh g^–1^ at cycle 200.

The overall specific capacities of the VISiCNT structures in set two of samples, ranging from 1550 to 3580 mAh g^–1^ (as shown in Figure 4a) are markedly higher than the 580–2460 mAh g^–1^ range observed in set one of the samples (Figure 3a). This improvement is attributed to the use of intentionally defective and shorter CNTs in the second set (with Raman *I*_D_/*I*_G_ ratios exceeding 1), as opposed to the high-quality CNTs (*I*_D_/*I*_G_ ≈ 0.35) used in set one. Defective CNTs, characterized by fractured structures and fragments, facilitate enhanced intercalation and diffusion of Si and Li ions for improved capacity.^36,58,59^ Consequently, both the CNT length and structural quality are critical determinants of the overall performance of the VISiCNT anodes. *In situ* CNT growth offers the advantage of directly tuning CNT height and defect density for optimum anode performance, eliminating the need for postsynthesis treatments (e.g., acid or plasma processing) that are typical of *ex situ* processes.^59,60^

The capacity decay rate and the specific capacity at cycle 15 as a function of CNT length (from 1 μm of SC/CNT/Si800 to 10 μm of EC/10CNTs/Si) are shown in Figure 4b. Although the SC/VS-CNTs/Si sample is excluded from this graph due to its unique nanostructure (comprising carbon-encapsulated nanoparticles and extremely short, sparse CNTs), the remaining samples display a trend in which the decay rate decreases from 5.47 to 2.93 mAh g^–1^·cycle^–1^ as the CNT length increases from 1 to 10 μm. Nevertheless, the specific capacity at cycle 15 remains substantially higher for electrodes incorporating shorter CNTs, as previously discussed. The slower capacity decay observed in longer CNT samples is likely due to the formation of a thinner conformal Si coating on the CNTs upon cycling. This phenomenon is attributed to the Si migration from the CNT tips toward their roots as a result of pulverization during cycling, as observed in Figure 5g,h and schematically depicted in Figure 5i). Thicker Si layers are prone to progressive fragmentation (electrochemical milling) during cycling, which continually disrupts the existing SEI and necessitates the formation of a new one on the freshly exposed surface.^31,54^ Therefore, a thinner, conformal Si coating on CNTs is favorable for establishing a stable SEI and ensuring efficient alloying/dealloying, owing to the shorter charge transfer distance. This result implies that both the CNT length and the corresponding Si thickness must be optimized in the fabrication of the VISiCNT structure for optimal battery performance. The relatively low decay rate observed in the SC/VS-CNTs/Si sample (∼3.5 mAh g^–1^·cycle^–1^) appears to stem from its distinctive nanostructured morphology, characterized by extremely short, sparse, and defect-rich CNTs, as confirmed by Raman analysis (Figure S6e).

Figure 4c presents the areal capacities and Coulombic efficiencies of the second set of samples. Because the SC/CNT/Si800 electrode contains a significantly thicker Si film (800 nm) than the other samples (200 nm Si), it delivers a higher areal capacity. The sample without CNTs (Cu/Si) exhibits the lowest areal capacity, whereas EC/2CNTs/Si shows the highest areal capacity among the electrodes with 200 nm Si. This improvement arises from the shorter CNT length (2 μm) in this sample compared with EC/10CNTs/Si and SC/5CNTs/Si, as discussed earlier. The SC/VSCNTs/Si electrode also demonstrates good capacity and better cycling stability. Since the EC/2CNTs/Si sample provides both higher areal and specific capacities, a CNT height of around 2 μm appears to be optimal for a 200 nm Si coating. Although the SC/VSCNTs/Si sample shows lower areal capacity than EC/2CNTs/Si, its specific capacity is slightly higher due to the negligible mass contribution of its very short (<0.5 μm) and sparse SC/VS-CNTs.

The maximum areal capacity of approximately 0.35 mAh cm^–2^ achieved for the sample with an 800 nm Si layer, is still below commercial requirements, which typically exceed 2 mAh cm^–2^. This suggests that Si films thicker than 4 μm will be necessary to reach practical capacity levels. Correspondingly, the CNT height will also need to be reoptimized for such thicker Si layers, which may be explored in our future work.

Beyond the initial cycles, all samples exhibit high Coulombic efficiencies exceeding 98%, with some samples even achieving efficiencies above 100%. This anomalously high efficiency may be due to the eventual release of previously trapped Li^+^ ions or the occurrence of secondary side reactions (Figure 4c). Detailed Coulombic efficiency values for both the first cycle and cycle 15 are provided in Table S3 of the SI. Postcycling electrochemical impedance spectroscopy (EIS) analysis (Figure 4d) was performed following the methodology reported by Guo and coworkers for carbon nanofiber/silicon anodes.^61^ The resulting Nyquist plots reveal a depressed semicircle (nonideal), followed by a diffusion tail at low frequencies. The semicircular region is influenced by the resistances of the SEI layer, the interfacial contact, and the charge transfer processes. The strong agreement between the impedance data and the cycling results emphasizes the benefits of the VISiCNT structure. Specifically, electrodes incorporating CNTs (EC/2CNTs/Si and EC/10CNTs/Si) exhibit lower impedance compared to the sample without CNTs (EC/Si), and electrodes with shorter CNTs show lower charge transfer resistance, corroborating the higher specific capacities reported in Figure 4a.

Figure 5a,b presents SEM images of the EC/10CNTs/Si sample before and after electrochemical cycling, respectively, revealing significant pulverization and crack formation as a result of cycling-induced stress. Quantitative analysis using ImageJ, as shown in Figure 5c, estimated the crack area to be approximately 19%. In comparison, Figure 5d,e displays the EC/2CNTs/Si samples before and after cycling, respectively. Image analysis of Figure 5f indicates a larger crack area of approximately 38%. This suggests that the use of shorter CNTs provides greater mechanical accommodation for the volumetric expansion/contraction of silicon during cycling, potentially contributing to the enhanced capacity observed in Si–CNT–CS incorporating shorter CNTs.

Cross-sectional SEM images in Figure 5g,h further illustrate the morphological evolution of the EC/2CNTs/Si sample, showing substantial silicon migration toward the CNT roots and an estimated volume expansion of ∼400% (from a thickness of ∼2 μm to ∼10 μm). This structural transformation is schematically represented in Figure 5i. Notably, despite the pronounced expansion, the active material in both the EC/10CNTs/Si and EC/2CNTs/Si samples remains relatively well adhered to the current collector, indicating good mechanical integrity. The cross-sectional SEM images in Figure 5g,h reveal that, after cycling, the Si evolves from a relatively thick film or aggregated chunks into a thinner, more conformal coating around the CNTs. This structural transformation is a key contributor to the enhanced electrochemical performance, as it reduces the effective Li-ion diffusion length, accelerates reaction kinetics, and promotes the formation of a more stable SEI. In combination with the uniform coating, this morphology helps alleviate mechanical stress and suppresses the pulverization typically associated with the 300%–400% volume expansion of Si anodes. In contrast, the EC/Si sample, which lacks CNT reinforcement, exhibits severe degradation of the silicon layer postcycling, as depicted in Figure 5j–l. This structural failure correlates with the rapid capacity fading observed beyond 50 cycles, as shown in Figure 4a, underscoring the critical role of CNTs in enhancing the structural stability and cycling performance of silicon-based electrodes.

## Conclusion

The successful growth of high-quality CNTs on thin Cu foil (10 μm) with precise control over CNT length and structural quality, followed by the subsequent fabrication of the VISiCNT structure, are unprecedented and can be seamlessly integrated into existing large-scale cell manufacturing processes. The *in situ* CNT growth process offers the unique advantage of directly tuning CNT height and defect density, both of which are critical parameters for optimizing battery performance. The carefully optimized VISiCNT anode structure achieves one of the highest anodic capacities of 3580 mAh g^–1^. Moreover, the achieved high-quality (*I*_D_/*I*_G_: 0.59) CNT growth rate of 21 μm/min on Cu foil below 450 °C demonstrates significant potential for large-scale roll-to-roll production of CNTs applicable to a wide range of fields. While this work clearly paves the way toward high anodic performance for high-energy-density batteries suitable for electric vehicles and energy storage systems, the approach can also be directly leveraged to design microsized lithium-ion batteries for microelectronic applications.

## Supplementary Material



## Notes

Weight measurement method, table of mass loading on the samples, SEM images of CNTs grown directly on Cu foil (set one samples), Raman spectrum of Si coating on CNTs, formation cycle and differential plot of set one samples, postcycling SEM images of set one samples, SEM images and Raman spectra of set two samples, and table of capacity values and related data (PDF)
