# Supplementary material for: Vertically Integrated Silicon–Carbon Nanotube Architectures for High-Capacity and Robust Lithium-Ion Battery Anodes
Source: ACS Appl Energy Mater. 2026 Mar 23;9(7):3818–28. doi: 10.1021/acsaem.5c03862 (PMC13080772; doi:10.1021/acsaem.5c03862)
Supplement: Supplementary file 1 [file ae5c03862_si_001.pdf]

Supporting Information of "Vertically Integrated Silicon-Carbon Nanotube Architectures for High-Capacity and Robust Lithium-Ion Battery Anodes"

Muhammad Ahmad[1,2*], Asim Mumtaz[3,4], Filipe Braga[5], Kai Yang[1,2], Peter Yates[4,6], Thomas P Shalvey[4], Oliver S. Hutter[4,7], Matthew Bilton[8], Jonathan D. Major[4], Ken Durose[4], Laurence J. Hardwick[5], S. Ravi P. Silva[1,2*]

[1]Advanced Technology Institute, School of Computer Science and Electronic Engineering, University of Surrey, Guildford, GU2 7XH

[2]Institute for Sustainability, University of Surrey, Guildford, GU2 7XH

[3]University of York, School of Physics, Engineering and Technology, Heslington, York, YO10 5DD

[4]University of Liverpool, Stephenson Institute for Renewable Energy, Department of Physics, Peach Street, Liverpool, L69 7ZF

[5]University of Liverpool, Stephenson Institute for Renewable Energy, Department of Chemistry, Peach Street, Liverpool, L69 7ZF

[6]The University of Manchester, Nancy Rothwell Building, Booth Street East, Manchester M1 7HL

[7]Northumbria University, Newcastle upon Tyne, NE1 8QH.

[8]SEM Shared Research Facility, University of Liverpool, Brownlow Street, Liverpool, L69 3GL

*Corresponding Author Email: muhammad.ahmad@surrey.ac.uk

*Corresponding Author Email: S.Silva@surrey.ac.uk

Weight Measurement Method

In this study, we highlight an often-overlooked challenge: accurately measuring the mass loading of ultra-lightweight active materials in electrodes. For example, Abdul-Rahman et al. reported an average mass loading of 265 µg/cm² with a large error of ±100 µg/cm² for their graphene-CNT structure on Cu foil.[1] One key issue is that the measurement error associated with the Cu discs outweighs the actual mass of the active material. For instance, we measured 20 Cu discs (50 µm thickness with a 1.13 cm² area) and obtained an average mass of 49.43 ± 0.27 mg. In contrast, the mass of CNTs grown on the same area to a height of 15 µm was approximately 0.13 mg, and that of a 200 nm Si layer was around 0.03 mg; both significantly lower than the ±0.27 mg uncertainty associated with the Cu discs. To overcome this limitation and improve accuracy, we measured the mass of each individual Cu disc before and after CNT growth and subsequent Si deposition, rather than relying solely on an average value. From these measurements, the average CNT mass was determined to be 0.009 ± 0.002 mg per µm of CNT height on each Cu disc. Likewise, the average mass of a 200 nm Si layer on a 1.13 cm² disc was calculated as 0.0328 ± 0.0013 mg, as detailed in Table S1, with the complete set of sample weight data provided in Table S2.

Table S1. Si mass measurements. A film of ***350 nm Si*** *was sputtered on 1.5 cm diameter Cu discs. The weight of the discs was individually measured before and after Si deposition.*

**Sample No.**	**Bare disc mass (mg)**	**Mass after Si deposition (mg)**	**Si Mass (mg)**
1	15.673	15.765	0.092
2	15.879	15.968	0.089
3	15.864	15.960	0.096
4	15.761	15.852	0.091
5	15.918	16.003	0.085
6	15.775	15.860	0.085
7	15.956	16.048	0.092
8	15.799	15.888	0.089
Average Si Mass		0.0899 ± 0.0035 mg	
Si Density		1452.52 mg/cm^3	
Vol. of 200 nm Si on 1.13 cm^2 Disc		2.26 × 10^{-5} cm^3	
Vol. of 800 nm Si on 1.13 cm^2 Disc		9.04 × 10^{-5} cm^3	
Mass of 200 nm Si on 1.13 cm^2 Disc		**0.0328 ± 0.0013 mg**	
Mass of 800 nm Si on 1.13 cm^2 Disc		**0.1313 ± 0.0051 mg**	
CNT Mass		**9.0 × 10^{-3} ± 0.002 mg/µm**	

Table S2. Mass loading of different samples.

Sample Name	CNT Length (μm)	CNT Mass (mg)	Si Mass (mg)	Total Mass (mg)
150CNTs	150	1.35	0	1.350
3CNTs	3	0.027	0	0.027
3CNTs/Si	3	0.027	0.033	0.060
15CNTs/Si	15	0.135	0.033	0.168
EC/10CNT/Si	10	0.09	0.033	0.123
EC/2CNTs/Si	2	0.018	0.033	0.051
EC/Si	-	0	0.033	0.033
SC/VS-CNTs/Si	< 0.5	0	0.033	0.033
SC/5CNTs/Si	5	0.045	0.033	0.078
SC/CNTs/Si800	1	0.009	0.131	0.140
Cu/Si	-	0	0.033	0.033

Set one of Samples

Figure S1 provides a detailed SEM visualization of different CNT samples, highlighting their structure and modifications. The 150CNTs (Figure S1(a, b)) sample exhibit dense, vertically aligned nanotubes of about 150 μm height, while the 3CNTs (Figure S1c) sample shows significantly shorter (~3 μm) CNTs. The 3CNTs/Si (Figure S1d) variation includes a 200 nm sputtered silicon layer on top of the CNTs.

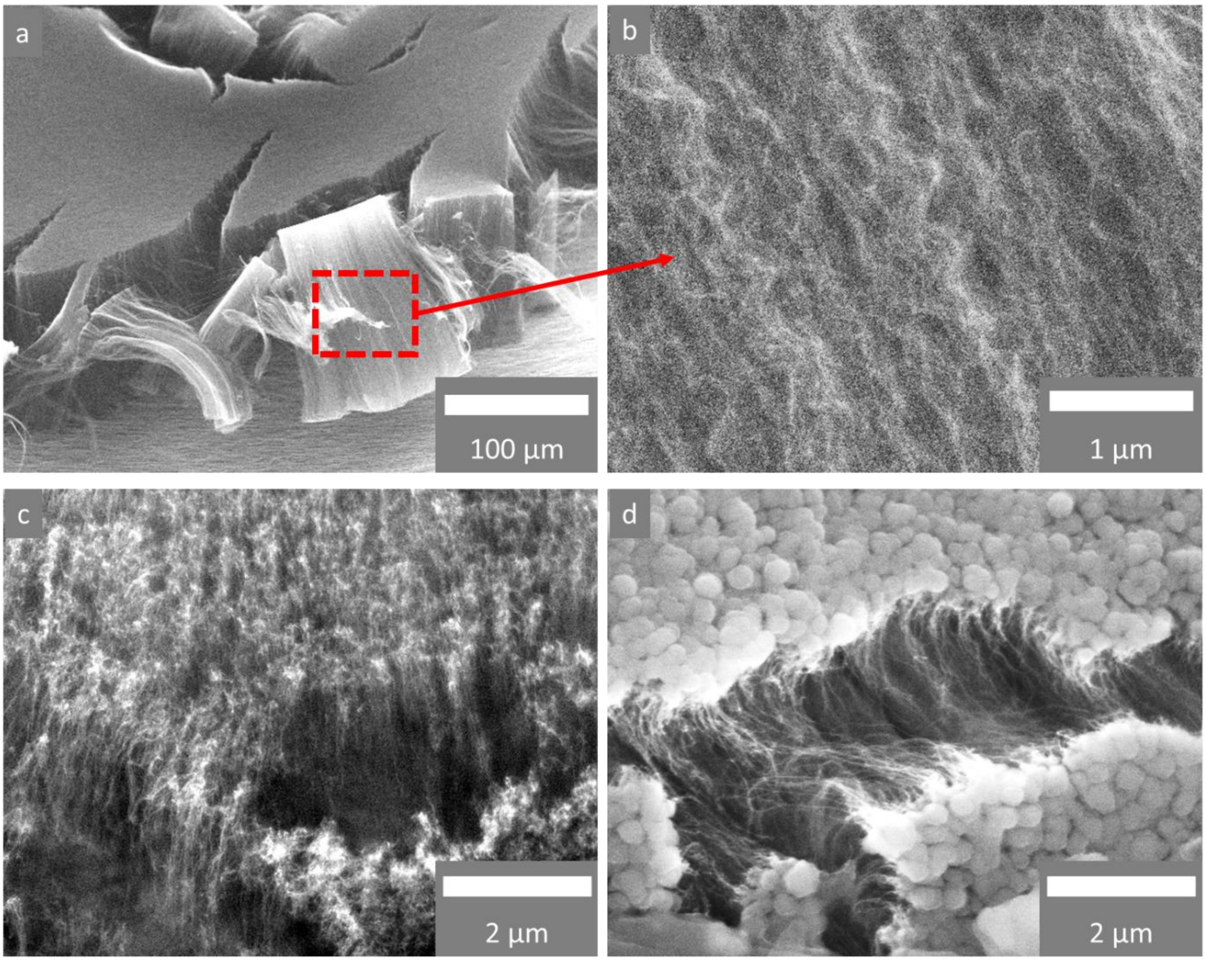

Figure S1. SEM images of CNTs grown directly on Cu foil using PTCVD. (a) The image showing vertically aligned CNTs of length ~150 µm (150CNTs), (b) High magnification image of 150CNTs sample showing dense CNTs. (c) vertically aligned CNTs of length ~3 µm grown on Cu foil (3CNTs). (d) The image showing 200 nm Si sputtered on top of 3 µm long CNTs (3CNTs/Si).

Figure S2 shows the SEM images of 15CNTs/Si sample. Figure S2a shows as grown vertically aligned CNTs of length ~15 µm and Figure S2 (b-d) shows images of these CNTs subsequently coated with 200 nm Si.

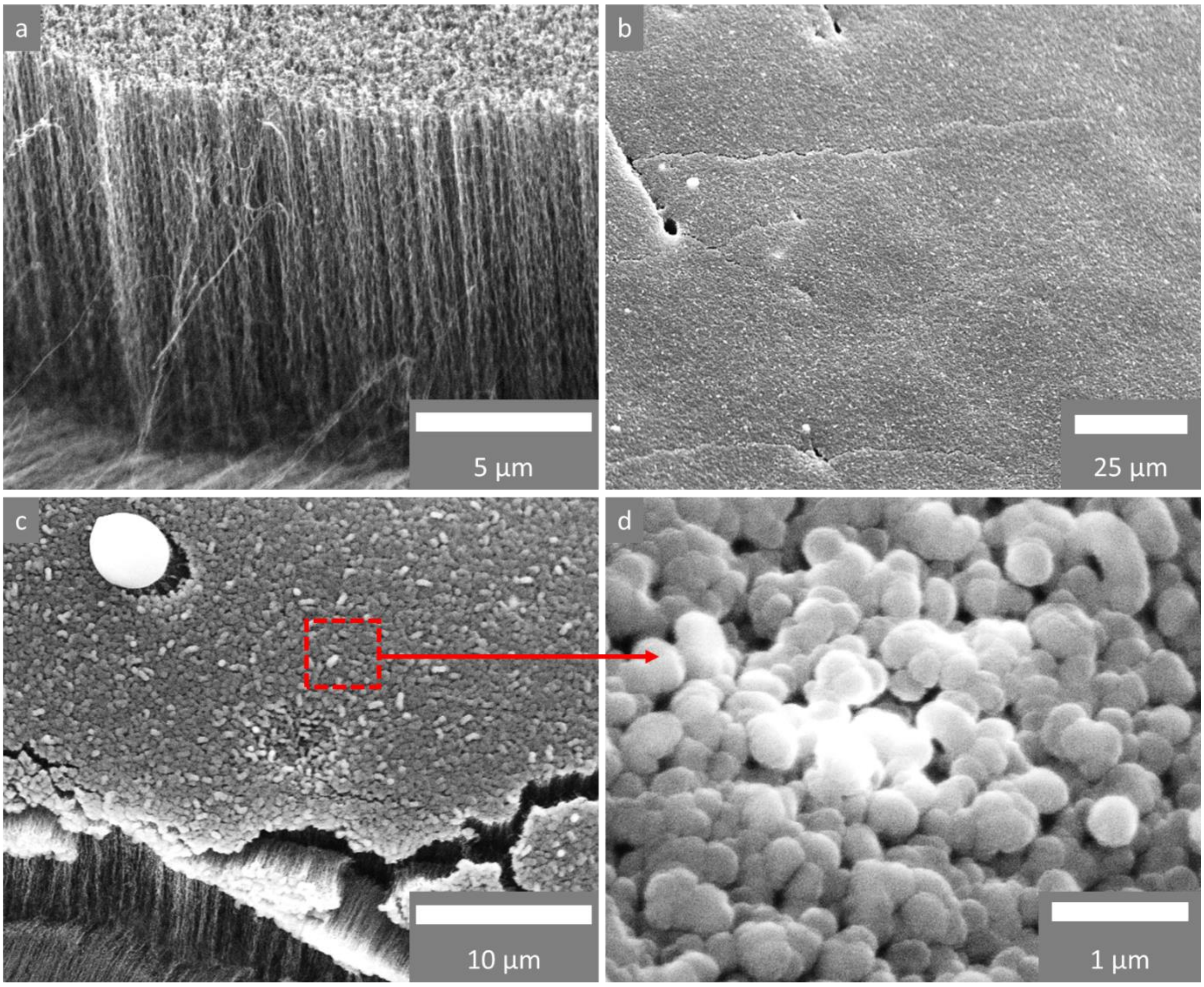

Figure S2. SEM images of Si coated CNTs of length 15 µm (15CNTs/Si) grown directly on Cu foil using PTCVD technique. (a) The image showing dense and vertically aligned CNTs grown on Cu foil, (b) 200 nm Si sputtered on top of the CNTs, (c) cross-sectional view of the Si coated CNTs, (d) high magnification top view of Si coated CNTs.

Figure S3 shows Raman spectrum of Si sputtered on CNTs. As, crystalline silicon has uniform bond angles and bond lengths, so it results in a sharp Raman peaks centred at 521 cm^{-1}. On the other hand, the Raman spectrum of Figure S3 exhibiting broad Raman band centred around 480 cm^{-1} resembles that of amorphous silicon which is less orderly in its arrangement with a wider array of bond angles, dangling bonds and bond energies.

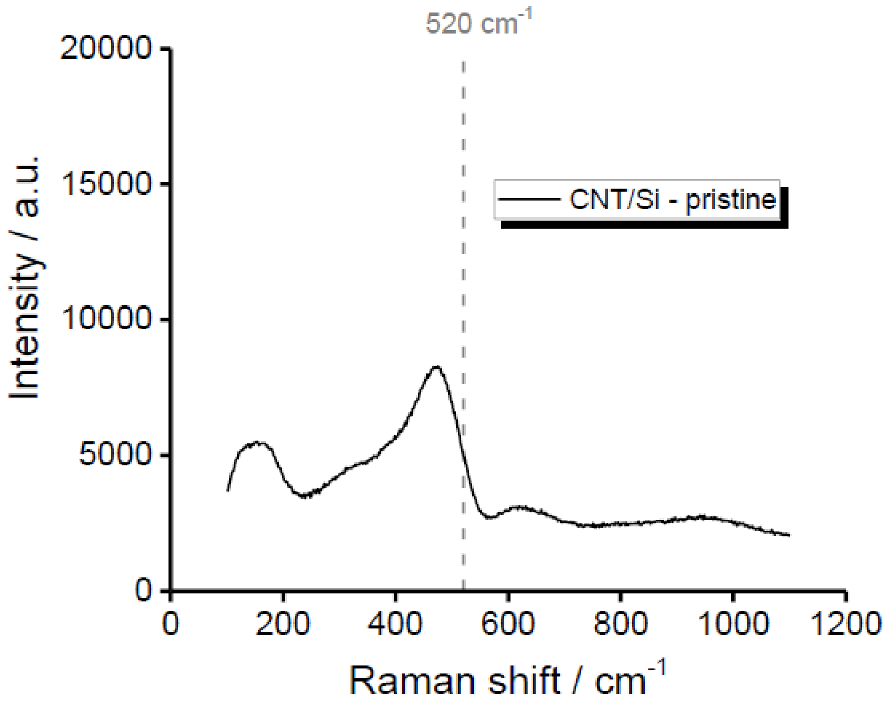

Figure S3. Raman spectrum of sputtered Si on CNTs showing a broad peak centred around 480 cm^{-1}, which indicates amorphous Si.

Figure S4 shows the formation cycle and differential plot of the first set of samples comprising 150CNTs, 3CNTs, 3CNTs/Si and 15CNTs/Si. In Figure S4a, 150CNTs show an extended voltage plateau (between 0.95 – 0.8 V) and much higher capacity of ~0.33 mA/cm^2 as compared with the 3CNTs (0.09 mA/cm^2). Whereas the capacity of Si coated CNT samples (3CNT/Si and 15CNT/Si) achieves ~0.24 mA/cm^2. The curves for 150CNTs and 3CNTs samples show voltage plateau centred at 0.9 V. The differential capacity plot (Figure S4b) exhibit a peak at approximately 0.9 V for 150CNTs, which is associated to the formation of the SEI layer, and consumes Li irreversibly.

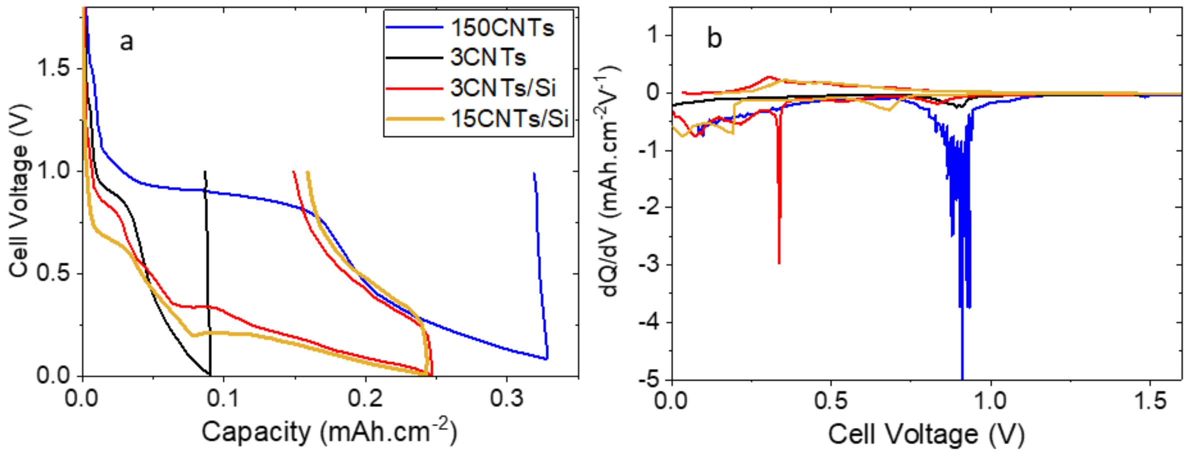

Figure S4. (a) The formation cycle performed at slower rate (C/20) shows much higher discharge capacity for 150CNTs than 3CNTs sample, whereas the VISiCNT structure exhibit high capacity of around 0.24 mAh.cm^{-2}. (b) The differential voltage plot showing a large peak at 0.9 V for 150CNT sample is associated with the formation of SEI layer.

Figure S5 shows SEM images of the cycled samples of set one, where pure CNTs of length 150 µm (150CNTs) and 3 µm (3CNTs) grown directly on Cu foil were used as anode. Deformation of 150 µm long CNTs due the compression cause by the cell assembly and densification effect caused by the liquid electrolyte can be observed in Figure S5 (a, b). Also, increase in the CNT diameter from 10-20 nm to 50-60 nm due to the SEI formation and irreversible Li trapping can be noted in high magnification SEM images. The key observation is that the CNTs remained firmly attached with the Cu foil during the cycling process.

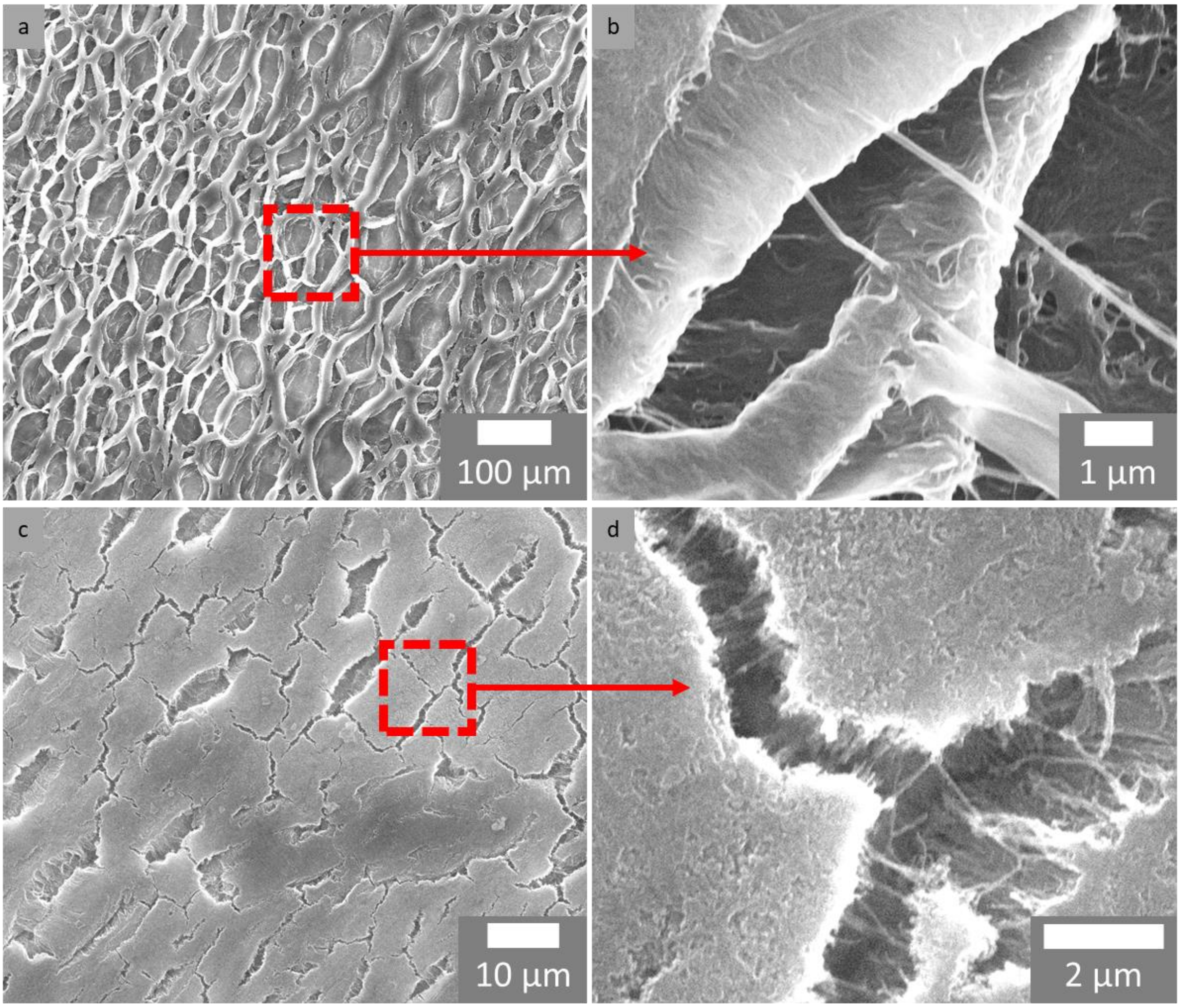

Figure S5. Post cycling SEM images of set one of CNT samples. (a, b) lower and higher magnification images of 150CNTs sample showing deformation of CNT alignment as well as increment of CNT diameters to 50-60 nm. (c, d) lower and higher magnification of 3CNTs sample. CNTs of all the samples remained well adhered to the Cu foil.

Set Two of Samples

Figure S6 shows SEM images and Raman spectra of the second set of samples where CNTs of relatively shorter length are grown directly on Cu foil using the PTCVD technique. Figure S6a shows the image of EC/10CNT/Si sample where CNT bundles of different heights ranging from 5-15 µm (10 µm is taken as average) can be observed. Figure S6b shows SEM image of SC/5CNT/Si sample where vertically aligned CNTs of ~5 µm height were grown. Figure S6c SEM image of SC/VS-CNTs/Si sample where CNT growth was performed only for 15 sec, resulting in very short and sparce CNTs of length less than 500 nm. Inset depicts the carbon encapsulation of the catalyst nanoparticles. Figure S6d SEM image of SC/CNT/Si-800 sample after CNT growth showing tangled CNTs of around 1 µm length. Figure S6e Raman spectra of selected samples showing higher $I_D/_G$ values (1, 1.11) because of the growth of defective CNTs as compared set one of samples.

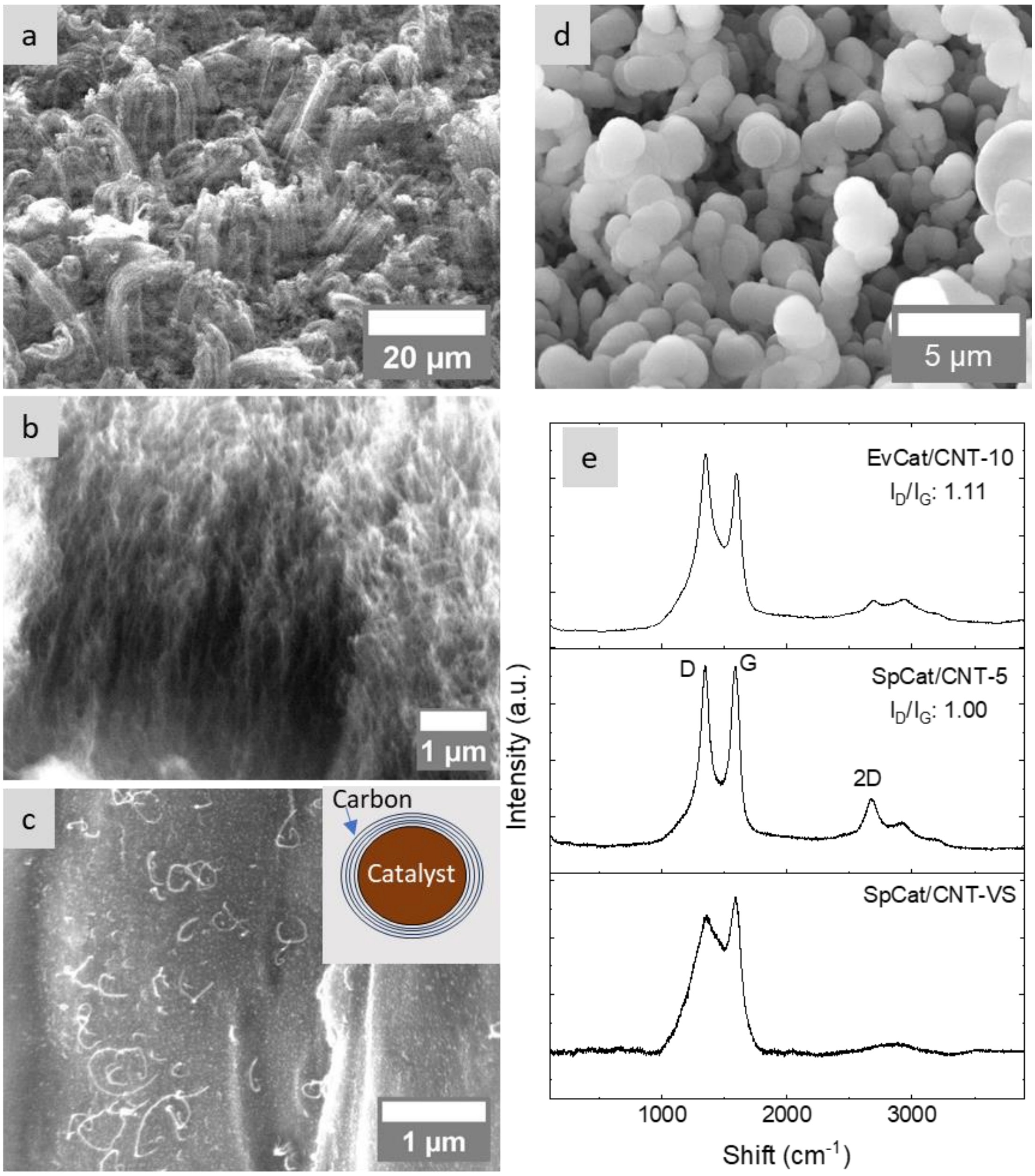

Figure S6. SEM images and Raman spectra of set two of samples. (a) SEM image of EC/10CNT/Si sample showing CNT bundles of varying height ranging from 5-15 µm (10 µm is taken as average). (b) SEM image of SC/5CNT/Si sample showing vertically aligned CNTs of ~5 µm height. (c) SEM image of SC/VS-CNTs/Si sample showing carbon encapsulated catalyst particles and just initiation of CNT growth with few CNTs of length less than 500 nm. Inset of the image depicts carbon encapsulation of the catalyst nanoparticles. (d) SEM image of SC/CNT/Si-800 sample after 800 nm Si sputtering on CNTs. (e) Raman spectra of selected samples showing higher $I_{D/G}$ values indicating more defects as compared to the spectra of set one of samples.

Table S3 contains that capacity values at cycle no. 15 and cycle no. 198 of second set of samples, and also the coulombic efficiency values.

Sample Name	Specific Capacity at Cycle 15 (mAh/g)	Specific Capacity at Cycle 198 (mAh/g)	Capacity Loss (%)	1st Cycle Coulombic efficiency (%)	Coulombic efficiency (%) at Cycle 15
EC/2CNTs/Si	3545	2706	24	85	99.3
EC/10CNTs/Si	1552	995	36	72	98.5
EC/Si	3120	1021	67	89.7	99.5
SC/VS-CNTs/Si	3586	2941	18	95	101.2
SC/5CNTs/Si	1643	1034	37	81	100
SC/CNT/Si800	2727	1731	37	91.3	99.7
Cu/Si	1675	464	72	89	99.4

References

1. Raji, A.-R.O., et al., *Lithium Batteries with Nearly Maximum Metal Storage.* ACS Nano, 2017. **11**(6): p. 6362-6369.
